# Noise-Induced Precursors of State Transitions in the Stochastic Wilson–Cowan Model

**DOI:** 10.1186/s13408-015-0021-x

**Published:** 2015-04-08

**Authors:** Ehsan Negahbani, D. Alistair Steyn-Ross, Moira L. Steyn-Ross, Marcus T. Wilson, Jamie W. Sleigh

**Affiliations:** School of Engineering, The University of Waikato, Hamilton, 3200 New Zealand; Waikato Clinical School, University of Auckland, Hamilton, 3204 New Zealand

**Keywords:** Wilson–Cowan model, Stochastics, Phase transition, Bifurcation, Critical slowing down, Hopf, Saddle-node, Turing, Spatio-temporal patterns

## Abstract

The Wilson–Cowan neural field equations describe the dynamical behavior of a 1-D continuum of excitatory and inhibitory cortical neural aggregates, using a pair of coupled integro-differential equations. Here we use bifurcation theory and small-noise linear stochastics to study the range of a phase transitions—sudden qualitative changes in the state of a dynamical system emerging from a bifurcation—accessible to the Wilson–Cowan network. Specifically, we examine saddle-node, Hopf, Turing, and Turing–Hopf instabilities. We introduce stochasticity by adding small-amplitude spatio-temporal white noise, and analyze the resulting subthreshold fluctuations using an Ornstein–Uhlenbeck linearization. This analysis predicts divergent changes in correlation and spectral characteristics of neural activity during close approach to bifurcation from below. We validate these theoretical predictions using numerical simulations. The results demonstrate the role of noise in the emergence of critically slowed precursors in both space and time, and suggest that these early-warning signals are a universal feature of a neural system close to bifurcation. In particular, these precursor signals are likely to have neurobiological significance as early warnings of impending state change in the cortex. We support this claim with an analysis of the *in vitro* local field potentials recorded from slices of mouse-brain tissue. We show that in the period leading up to emergence of spontaneous seizure-like events, the mouse field potentials show a characteristic spectral focusing toward lower frequencies concomitant with a growth in fluctuation variance, consistent with critical slowing near a bifurcation point. This observation of biological criticality has clear implications regarding the feasibility of seizure prediction.

## Introduction

The underlying mechanism of an abrupt state transformation in a multi-stable dynamical system is well described using bifurcation theory. In a close vicinity to a state change or *tipping point*, dynamical systems exhibit increased susceptibility and fragility, which manifests as amplification and prolongation of the system response to intrinsic or experimentally induced perturbations [1]. This phenomenon, known as critical slowing down, is accompanied by increased variance [2] and higher autocorrelations [3, 4] of state fluctuations, and growth in spectral power at characteristic frequencies. Anticipation of an imminent tipping point mandates detection of its early-warning signals to permit timely intervention, particularly when the transition is unwelcome (e.g., species collapse) or pathological (e.g., seizure onset).

Identifying empirical indicators of an impending state transition is an area of active research across many disciplines including population ecology [5, 6], climate change [3], high-voltage engineering [7], and human behavior [8]. The notion of an abrupt phase transition, and its attendant critical signatures, has also been applied to *in vivo* and *in vitro* biological neural systems, and to simplified mathematical models of these.

Researchers have reported increased fluctuation power and slowed time-scales prior to the firing of action potentials in a squid giant axon [9], and in simplified point models of resonator and integrator neuron types [10]. Similar fluctuation surges in electrical activity have been observed in intra-cell recordings of the prelude to the down-to-up state transition for a rat neuron emerging from anesthesia [11]; in ECoG recordings during the period preceding emergence of synchronized epileptic seizure events [12]; and in EEG recordings during the natural or drug-induced switching of large-scale brain activity due to onset of sleep or anesthesia [13]. Although most work has focused on the temporal properties of the fluctuations, some researchers have also identified enhanced *spatial* correlations near a bifurcation point, for example near the transition between slow-wave sleep and REM [14]; and in a 1-D mean-field model of a cortex near the anesthetic critical point [15].

Our goal in the present paper is to examine critical slowing phenomena within the context of the classic Wilson–Cowan (W–C) continuum model of neural population dynamics [16, 17]. In recent work [18] we have examined the close approach to cortical phase transitions in a mature mean-field model (containing synaptic response functions, axonal wave equations, gap-junction diffusion, somatic integration) expressed as a moderately complicated set of coupled differential equations that require a minimum of 8 to 14 system variables (depending on the assumed symmetries in the couplings between the excitatory and inhibitory neural populations), making numerical and analytic manipulations unwieldy. The attraction of the W–C continuum model is its simplicity: with only two system variables (rather than 8, or 14, or more), it is possible to explore the close approach to bifurcation in a spatially extended neural model at relatively little analytic or computational cost. It is our hope that the present work might serve as a useful tutorial testbed that invites other researchers to begin investigating criticality in a simplified neural context. Although we have made extensive use of small-noise Ornstein–Uhlenbeck (O–U) theory in our previous studies, to our knowledge this is the first time this predictive and quantitative technique has been applied to the W–C model.

Critical transitions are mediated by specific bifurcation classes, so we are motivated to examine the signs of critical slowing exhibited by each class of bifurcation that is accessible to the spatially extended Wilson–Cowan model. The specific bifurcations of interest are saddle-node, Hopf, Turing, and mixed-mode Turing–Hopf interactions.

We must acknowledge the rich and extensive literature discussing temporal and spatial bifurcations in Wilson–Cowan (and W–C-like) mean-field neural models. For example, Ermentrout and Cowan [19], and more recently Bressloff [20], have explored diffusion-driven Turing instabilities in the W–C model supporting formation of stationary activity patterns that have been likened to visual hallucinations. Laing and Troy [21] derived stability conditions for so-called multi-bump neural activity patterns; Coombes and Laing [22] introduced delays into the W–C model and demonstrated Hopf and saddle-node bifurcations as well as bursting behavior. Close approach to the Hopf instability induces spectral growth in specific EEG frequency bands, and this idea has been investigated in an anesthesia context recently by Hutt [23] and by Hindriks and van Putten [24], with the latter work based on a thalamocortical model by Robinson et al. [25]. Hutt et al. [26–28] have studied the impact of noise on spatio-temporal instabilities in neural fields containing synaptic and transmission delays, and has established conditions for emergence of Hopf and Turing instabilities, identifying the growth of fluctuation variance on approach to bifurcation with critical slowing, and showing that under certain conditions, noise can delay Turing onset. We will revisit the latter point in the Discussion.

The paper is organized as follows: Sect. 2 describes the spatially extended Wilson–Cowan model. We locate its homogeneous stationary state and identify parameter settings that cause the state to lose stability via distinct bifurcations: saddle-node, Hopf, or Turing. We introduce additive noise, and detail a theoretical technique (Ornstein–Uhlenbeck linearization), based on the subthreshold eigenvalue structure of the steady state, that accurately predicts the statistical properties of the noise-induced fluctuations. The results of a series of stochastic numerical experiments are presented in Sect. 3 where we demonstrate critically slowed fluctuations—emerging as patterns in time and space—for close approach to bifurcation; these emergent patterns are concordant with O–U theoretical projections. To confirm relevance of critical slowing to neuroscience, we describe electrophysiological recordings taken from slices of mouse-brain tissue that have been chemically conditioned to intermittently reenter a seizure-like state. Section 4 concludes with a discussion of the apparent universality of critical slowing prior to bifurcation, and its potential usefulness as an early-warning biosignal of impending state change in neural systems.

## Mathematical Model

The Wilson–Cowan (W–C) model describes the average spike frequency of a neural population as a function of continuous time. The fundamental assumption is that brain activity can be described in terms of interactions between excitatory and inhibitory populations, so the state variables for the model are *E* and *I*, the excitatory and inhibitory spike-rates, respectively. Based on known neuroanatomy, Wilson and Cowan assumed a random, dense connectivity between individual cells, allowing at least one connection between any two cells in the network.

Wilson and Cowan neglected spatial interactions in their original 1972 paper [16] in order to investigate temporal dynamics of a localized cortical column; they then extended their model to spatially distributed neural populations in their 1973 paper [17], and modeled 1-D rods and 2-D sheets of cortical and thalamic tissue.

In this section we introduce the W–C model equations in both their spatially homogeneous and their spatially varying 1-D forms, define their stationary states, and perform a linear stability analysis about these stationary states in Fourier (wavenumber *q*) domain to extract the eigenvalue-vs.-*q* dispersion curves, enabling us to identify potential bifurcations points at which stability of a given stationary state is predicted to disappear. We then introduce stochasticity into the model by adding small-amplitude spatio-temporal white noises, and compute a set of subthreshold fluctuation statistics (autocorrelations, temporal and spatial spectral densities) that allow us to quantify the expected alterations in fluctuation properties as the W–C model closely approaches a given critical point. We then detail the algorithm used to numerically integrate the stochastic W–C equations in one spatial dimension.

### Simplified Wilson–Cowan Equations

In 1999 Wilson presented a simplified form of neural equations [29] describing the collective behavior of cortical tissue in terms of a discrete *network* of laterally connected localized aggregates of *E* and *I* populations representing cortical columns. The simplified equations are

1 $$ \begin{aligned} \tau_{E} \frac{dE(x,t)}{dt}={}&{-}E(x)\\ &{}+\mathcal{S}_{E} \biggl( \sum_{y}w_{EE}(x-y)E(y)- \sum_{y}w_{IE}(x-y)I(y)+P \biggr), \\ \tau_{I} \frac{dI(x)}{dt}={}&{-}I(x)\\ &{}+\mathcal{S}_{I} \biggl( \sum_{y}w_{EI}(x-y)E(y)-\sum _{y}w_{II}(x-y)I(y)+Q \biggr), \end{aligned} $$

where the $w_{jk}$ are synaptic strengths *from* population *jto* population *k* (e.g., $w_{IE}$ is the $I\rightarrow E$ connection strength). Note that the discrete summations actually represent spatial convolutions. For our theoretical work we choose to return these network equations to continuum form,

2 $$ \begin{aligned} \tau_{E} \frac{\partial}{\partial t} E(x,t) &=-E(x,t)+ \mathcal{S}_{E} \bigl[ w_{EE}(x)\otimes E(x,t) - w_{IE}(x)\otimes I(x,t) +P \bigr], \\ \tau_{I} \frac{\partial}{\partial t} I(x,t) &=-I(x,t)+ \mathcal{S}_{I} \bigl[ w_{EI}(x) \otimes I(x,t) - w_{II}(x)\otimes I(x,t) +Q \bigr], \end{aligned} $$

where $E(x)$ and $I(x)$ are the mean firing rate of neurons at position *x* in (ms)^−1^, $\tau_{E}$ and $\tau_{I}$ are the relaxation time-constants of each population (in ms), and *P*, *Q* (in mV) are the external voltage inputs entering each population. Here ⊗ represents a 1-D spatial convolution defined as

3 $$ \begin{aligned} f_{1}(x)\otimes f_{2}(x)\equiv \int_{-\infty}^{\infty}f_{1}\bigl(x' \bigr)\cdot f_{2}\bigl(x-x'\bigr)\,dx' \end{aligned} . $$

The four $w_{jk}$ ($j,k\in\{E, I \}$) in Eqs. (2) are connectivity functions that define the density (strength per unit length; units mV⋅ms/μm) of the synaptic coupling between and within populations. The coupling strength is assumed to decay exponentially with distance:

4 $$ w_{jk}(x)=\frac{b_{jk}}{2\sigma_{jk}}\exp\bigl(-\vert x\vert / \sigma_{jk}\bigr), $$

where $b_{jk}$ (in mV⋅ms) is the maximum synaptic coupling strength between populations *j* and *k*, and $\sigma_{jk}$ (in μm) is the space constant that defines the spatial extent of connectivity. (This form of normalization ensures that $\int_{-\infty}^{\infty }w_{jk}(x)\,dx=b_{jk}$ and is particularly useful when simplifying Eq. (2) to its spatially homogeneous limit (see Sect. 2.2).) Wilson [29] used a Naka–Rushton function to map from voltage to firing rate, but we elect to use the sigmoidal function,

5 $$ \begin{aligned} S_{j}(v)=\frac{S_{j}^{\max}}{1+\exp[-a_{j}(v-\theta_{j})]} ,\quad j\in\{I,E\}, \end{aligned} $$

where *θ* (in mV) is the threshold voltage for half-maximum firing, *a* (in (mV)^−1^) sets the sigmoid slope at threshold, and $S_{j}^{\max}$ (in (ms)^−1^) is the maximum firing rate.

### Homogeneous Wilson–Cowan Limit

As the simplest reference case, we investigate a homogeneous cortex in which the population firing rates are independent of position, so that $E(x,t)\rightarrow E(t)$, $I(x,t)\rightarrow I(t)$, and the convolution integrals in Eqs. (2) collapse to simple scaling of the population activities:

6 $$ \begin{aligned} \tau_{E} \frac{ d E}{ dt} & =-E+ \mathcal{S}_{E} (b_{EE} E- b_{IE} I+P), \\ \tau_{I} \frac{ d I}{ dt} & =-I+\mathcal{S}_{I} (b_{EI} E- b_{II} I+Q). \end{aligned} $$

While keeping the parameter values biologically plausible and broadly similar to those used in ref [30], we fine-tuned the sigmoidal parameters ($S_{E,I}^{\max}$, $a_{E,I}$, $\theta_{E,I}$) to generate at least one non-monotone nullcline; and the *P*, *Q* voltage inputs were selected to give a multi-root region in the steady-state distribution curve. See Table 1 for parameter values, and Sect. 2.4 for the definition of nullclines and steady-state diagram.

**Table 1:** **Symbol definitions and parameter values for 1-D Wilson–Cowan model**

Symbol	Description	Value (homogeneous)	Value (space dependent)	Unit
*E*, *I*	Excitatory and inhibitory firing rates			(ms)^−1^
$S_{E}$, $S_{I}$	Sigmoid functions			(ms)^−1^
$\tau_{E,I}$	*E* and *I* time constants	10, 8	10, 8	ms
$b_{EE, EI, IE, II}$	Synaptic coupling strength	18, 10, 10, 0	18, 10, 19, 0	mV⋅ms
$\sigma_{EE}$	*E*→*E* space constant	50	43, 50	μm
$\sigma_{EI}$	*E*→*I* space constant	110	[42–148.5]	μm
$\sigma_{IE}$	*I*→*E* space constant	110	[42–148.5]	μm
$\sigma_{II}$	*I*→*I* space constant	20	20	μm
$S_{E,I}^{\max}$	Maximum firing rate	0.1, 0.15	0.1, 0.15	(ms)^−1^
$a_{E,I}$	Sigmoid slope at threshold	9	9	(mV)^−1^
$\theta_{E,I}$	Half-maximum firing threshold	2.2	2.2	mV
*P*, *Q*	Exogenous voltage inputs	[0.9–3.3], 1.35	[0.9–3.3], 1.35	mV

### Space-Dependent Wilson–Cowan Model of 1-D Cortical Rod

The space-dependent integro-differential form of the Wilson–Cowan model of a 1-D “cortical rod” of length *L* can be directly derived from Eqs. (2),

7 $$\begin{aligned} \begin{aligned} \tau_{E}\frac{\partial}{\partial t}E(x,t) ={}&{-}E(x, t)+S_{E} \biggl[b_{EE}\int_{-L/2}^{L/2} E\bigl(x',t\bigr) n_{EE}\bigl(x-x'\bigr) \,dx' \\ &{}-b_{IE}\int_{-L/2}^{L/2} I \bigl(x',t\bigr) n_{IE}\bigl(x-x'\bigr) \,dx'+P \biggr], \\ \tau_{I}\frac{\partial}{\partial t}I(x,t) ={}&{-}I(x, t)+S_{I} \biggl[b_{EI}\int_{-L/2}^{L/2} E \bigl(x',t\bigr) n_{EI}\bigl(x-x'\bigr) \,dx' \\ &{}-b_{II}\int_{-L/2}^{L/2} I \bigl(x',t\bigr) n_{II}\bigl(x-x'\bigr) \,dx' +Q \biggr], \end{aligned} \end{aligned}$$

where *L* is the length of integration domain, and $n_{jk}$ is defined as

8 $$ n_{jk}\bigl(x-x'\bigr)=\frac{1}{2\sigma_{jk}} \exp\bigl(-\bigl\vert x-x'\bigr\vert /\sigma_{jk} \bigr),\quad (j, k)\in\{E, I\}. $$

Assuming that *L* is much greater than the length of spatial spread of excitatory and inhibitory connections (i.e., $L\gg\sigma_{ij}$, $i,j\in \{E,I\}$) the range of integration in (7) can be extended to ±∞ with negligible error. We define $\phi_{Ek}$ and $\phi_{Ik}$ as excitatory and inhibitory input fluxes in (ms)^−1^ to population of type *k* as,

9 $$ \begin{aligned} \phi_{Ek}(x,t)&=\int_{-\infty}^{\infty} E\bigl(x',t\bigr) n_{Ek}\bigl(x-x'\bigr) \,dx', \\ \phi_{Ik}(x,t)&=\int_{-\infty}^{\infty} I \bigl(x',t\bigr) n_{Ik}\bigl(x-x'\bigr) \,dx',\quad k\in\{E,I\}. \end{aligned} $$

Then $\phi_{Ek}(x,t)$, $\phi_{Ik}(x,t)$ obey (see the Appendix for a proof):

10 $$ \begin{aligned} \biggl(\varLambda_{Ek}^{2}- \frac{\partial^{2}}{\partial x^{2}} \biggr) \phi _{Ek}(x,t)&=\varLambda_{Ek}^{2} E(x,t), \\ \biggl(\varLambda_{Ik}^{2}-\frac{\partial^{2}}{\partial x^{2}} \biggr) \phi _{Ik}(x,t)&=\varLambda_{Ik}^{2} I(x,t), \end{aligned} $$

where $\varLambda_{jk}=1/\sigma_{jk}$ is the inverse length-scale for connections. These wave equations for $\phi_{Ek, Ik}$ describe propagation of flux activity from distant excitatory and inhibitory populations into type-*k* synaptic inputs of the cortical rod. We introduce the flux variables here in order to compute a wavenumber-dependent Jacobian matrix $\tilde {\mathbf{J}}(q)$ (see Eq. (15)), and hence extract the eigenvalue-vs.-*q* dispersion curves for the space-dependent W–C cortex from which we can identify potential instability bifurcation points. (However, for our numerical simulations, we solve the integro-differential equations (2) directly; see Sect. 2.8 for further details.)

The equations for the 1-D space-dependent Wilson–Cowan neural continuum can be written,

11 $$ \begin{aligned} \frac{\partial}{\partial t}E(x,t) &= \bigl[-E(x, t)+S_{E}\bigl(b_{EE} \phi _{EE}(x,t)-b_{IE} \phi_{IE}(x,t)+P\bigr) \bigr]/\tau_{E} \\ &\equiv B_{1}(E,\phi_{EE},\phi_{IE}), \\ \tau_{I}\frac{\partial}{\partial t}I(x,t) &= \bigl[-I(x, t)+S_{I} \bigl(b_{EI} \phi_{EI}(x,t)-b_{II} \phi_{II}(x,t)+Q\bigr) \bigr]/\tau_{I} \\ &\equiv B_{2}(I,\phi_{EI},\phi_{II}), \end{aligned} $$

with the four long-range $\phi_{jk}$ fluxes obeying the partial differential equations (10).

### Steady States of Deterministic System

Following [15], we assume that the cortical rod normally operates close to a homogeneous equilibrium state with uniform firing rates ($E^{\mathrm{o}}$, $I^{\mathrm{o}}$). For the deterministic model of Eq. (11), the equilibrium points are defined by setting all space- and time-derivatives to zero, and replacing the $E(x,t)$ and $I(x,t)$ firing rates with their fixed-point values, independent of time and space,

12 $$ \begin{aligned} E^{\mathrm{o}}&=S_{E} \bigl(b_{EE} \phi_{EE}(x,t)-b_{IE} \phi_{IE}(x,t)+P\bigr), \\ I^{\mathrm{o}}&=S_{I}\bigl(b_{EI} \phi_{EI}(x,t)-b_{II} \phi_{II} (x,t)+Q\bigr). \end{aligned} $$

**Fig. 1:**
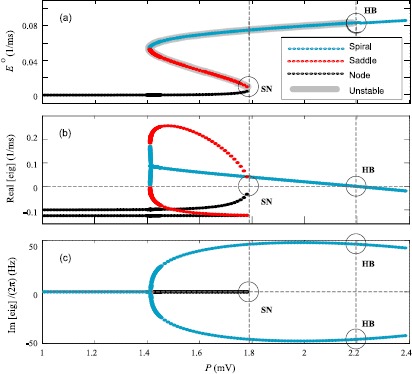
Steady-state distribution and eigenvalues of homogeneous Wilson–Cowan cortex. **(a)** Steady-state excitatory firing rate ($E^{\mathrm{o}}$) as a function of external excitatory input voltage *P*. **(b)** The real part of the eigenvalues corresponding to steady-state diagram of the model, determining stability and type of steady states. **(c)** The scaled imaginary part of eigenvalues, specifying the stationary oscillatory behavior of the system in Hz. Saddle-node (SN) and Hopf (HB) bifurcations switch the stability of the steady state near $P \approx1.7892426576\mbox{ and 2.1971513755 mV}$, respectively

Noting that at steady state, excitatory and inhibitory fluxes ($\phi _{Ek}$ and $\phi_{Ik}$) are equal to steady-state excitatory and inhibitory firing rates $E^{\mathrm{o}}$ and $I^{\mathrm{o}}$, we obtain the *nullcline* equations:

13 $$ \begin{aligned} E^{\mathrm{o}}&=\mathcal{S}_{E} \bigl(b_{EE}E^{\mathrm{o}}-b_{IE}I^{\mathrm{o}}+P \bigr), \quad \text{$E$-nullcline}, \\ I^{\mathrm{o}}&=\mathcal{S}_{I}\bigl(b_{EI}E^{\mathrm{o}}-b_{II}I^{\mathrm{o}}+Q \bigr), \quad \text{$I$-nullcline} \end{aligned} $$

whose intersections locate the ($E^{\mathrm{o}}$, $I^{\mathrm{o}}$) steady state. Figure 1(a) shows the distribution of steady states as a function of excitatory drive *P* for the parameter values of Table 1. We observe both single- and multi-root regions, with bifurcation points predicted when the steady states lose stability.

### Linear Stability Analysis of Deterministic Model

Linear stability analysis of the deterministic space-independent cortex allows us to predict the conditions under which temporal and spatial instabilities might emerge. We linearize Eqs. (11) by imposing a small perturbation $\hat{Z}$ around homogeneous stationary state $Z^{\mathrm{o}}$,

14 $$ Z(x,t)\rightarrow Z^{\mathrm{o}}+\hat{Z}(x,t),\quad Z \in\{E, I, \phi_{Ek}, \phi _{Ik}\} . $$

Here $\hat{Z}(x,t)=\delta_{z} \mathrm{e}^{\lambda t} \mathrm{e}^{iqx}$ has amplitude $\delta_{z}$, temporal evolution $\mathrm{e}^{\lambda t}$, and spatial mode $\mathrm{e}^{iqx}$ at wavenumber *q*. Substituting (14) in Eqs. (11) and Taylor-expanding to first-order, we obtain the linearized Wilson–Cowan model,

$$\begin{aligned} \frac{d}{d t}\tilde {\mathbf{u}}(t)=\tilde {\mathbf{J}}(q)\cdot \tilde {\mathbf{u}}(t), \end{aligned}$$

where

$$\tilde {\mathbf{u}}(t)=\left [ \begin{array}{@{}c@{}} \delta_{E} \mathrm{e}^{\lambda t}\\ \delta_{I} \mathrm{e}^{\lambda t} \end{array} \right ] $$

is the perturbation vector, and

15 $$ \begin{aligned} \tilde {\mathbf{J}}(q)=\left [ \begin{array}{@{}c@{\quad }c@{}} \frac{\partial B_{1}}{\partial E}+\frac{\partial B_{1}}{\partial\phi _{EE}}\frac{\varLambda^{2}_{EE}}{(\varLambda^{2}_{EE}+q^{2})}& \frac{\partial B_{1}}{\partial\phi_{IE}}\frac{\varLambda^{2}_{IE}}{(\varLambda^{2}_{IE}+q^{2})}\\ \frac{\partial B_{2}}{\partial\phi_{EI}}\frac{\varLambda^{2}_{EI}}{(\varLambda ^{2}_{EI}+q^{2})}& \frac{\partial B_{2}}{\partial I}+\frac{\partial B_{2}}{\partial\phi_{II}}\frac{\varLambda^{2}_{II}}{(\varLambda^{2}_{II}+q^{2})} \end{array} \right ] \end{aligned} $$

is the Jacobian matrix that is to be evaluated at stationary states ($E^{\mathrm{o}}$, $I^{\mathrm{o}}$); the *q*-dependent eigenvalues of this matrix determine system stability.

We analyze the space-independent case by setting $q=0$, and plotting the eigenvalue spectrum as a function of excitatory voltage drive *P*; see Fig. 1(b), (c). Two bifurcation types are evident: emergence of multiple states via *saddle-node* (SN) bifurcation, and loss of stability of the top branch via *Hopf* bifurcation (HB).

### Dispersion Curves of Space-Dependent Model

**Fig. 2:**
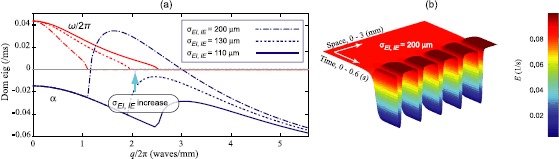
Prediction of Turing pattern formation in 1-D Wilson–Cowan cortex. **(a)** Dispersion curves corresponding to $P=2.34\mbox{ mV}$. The real and imaginary (scaled by 2*π*) parts of dominant eigenvalues are plotted versus wavenumber *q* for three different values of inhibitory synaptic range constants $\sigma_{EI, IE}$. Turing pattern is formed when the *α*-curve has positive excursions. **(b)** Corresponding color-coded spatio-temporal plot of 1-D cortex displaying the emergence of Turing pattern evolving from initial state of $E\approx0.0859\mbox{ 1/ms}$ corresponding to $P=2.34\mbox{ mV}$ and $\sigma_{EI,IE}=200\text{ μm}$

**Fig. 3:**
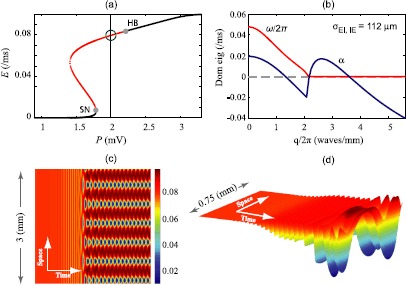
Emergence of Turing–Hopf mixed-mode oscillations in 1-D Wilson–Cowan cortex. **(a)** Steady state distribution with a selected point at $P=2\mbox{ mV}$ indicated by *green circle*. The system has a Hopf instability here. **(b)** Dispersion curve with synaptic range constant set to $\sigma_{EI,IE}=112\text{ μm}$ (see Table 1 for other parameter values) predicts a Turing instability at spatial frequency $q\simeq2.62\mbox{ waves/mm}$ and a temporal instability of frequency $f\simeq47\mbox{ Hz}$ (see the $\omega/2\pi$ value at $q=0$ axis on *dispersion curve*). **(c)** Bird’s-eye view and **(d)** 3-D spatio-temporal graphs demonstrate spatio-temporal evolution of 1-D network and emergence of mixed-mode Turing–Hopf oscillations in space and time

To explore the stability characteristics of the spatially extended 1-D model, we plot the distribution of *q*-dependent eigenvalues of the $\tilde {\mathbf{J}}(q)$ matrix. In Figs. 2(a) and 3(b) we plot the real and imaginary parts of eigenvalues at a selected steady state as a function of scaled wavenumber $q/2\pi$ to define the dispersion curve. Expressing the dominant eigenvalue as $\lambda=\alpha\pm\mathrm{j}\omega $, we identify the real part Re$(\lambda)\equiv\alpha$ as the damping rate, and Im$(\lambda)\equiv\omega$ as the oscillatory component. Thus instability at a particular wavenumber *q* is predicted if $\alpha(q)$ goes positive, in which case the oscillatory component will have spatial frequency $\omega(q)/2\pi$.

### Stochastic Model and Subthreshold Fluctuation Statistics

We can formulate a stochastic version of the Wilson–Cowan 1-D cortex by adding white-noise perturbations to the right hand side of Eqs. (11):

16 $$ \begin{aligned} \tau_{E}\frac{\partial}{\partial t}E(x,t) ={}&{-}E(x, t)+S_{E} \bigl(b_{EE} \phi _{EE}(x,t)-b_{IE} \phi_{IE}(x,t)+P \bigr)\\ &{}+c_{1}\xi_{1}(x, t), \\ \tau_{I}\frac{\partial}{\partial t}I(x,t) ={}&{-}I(x, t)+S_{I} \bigl(b_{EI} \phi _{EI}(x,t)-b_{II} \phi_{II}(x,t)+Q\bigr)\\ &{}+c_{2}\xi_{2}(x, t), \end{aligned} $$

where $c_{1,2}$ are scaling constants that ensure that the fluctuations are small, and $\xi_{1,2}$ are a pair of independent zero-mean, Gaussian-distributed spatio-temporal white-noise sources [15],

17 $$ \begin{aligned} \bigl\langle \xi(x,t)\bigr\rangle =0 , \quad \bigl\langle \xi_{m}(x,t)\xi_{n}\bigl(x',t' \bigr)\bigr\rangle =\delta _{mn}\delta\bigl(x-x'\bigr)\delta \bigl(t-t'\bigr) \end{aligned} , $$

where $\delta_{mn}$ is the dimensionless Kronecker delta, $\delta(\cdot )$ is the Dirac delta with a dimensionless total area under its curve, and the $\langle\cdot\cdot\cdot\rangle$ represents the ensemble average over space and time. The noise terms represent naturally occurring stochasticity in neural systems arising from (i) random spatio-temporal changes of neuron properties, and (ii) continuous random bombardments of neural membrane and ion channels originating from other neural populations.

Following a method similar to that presented in Sect. 2.5 for the deterministic model, linearization of the stochastic form results in

18 $$ \begin{aligned} \frac{\partial}{\partial t} \left [ \begin{array}{@{}c@{}} \hat{E}(x,t)\\ \hat{I}(x,t) \end{array} \right ]= \tilde {\mathbf{J}}(q) \left [ \begin{array}{@{}c@{}} \hat{E}(x,t)\\ \hat{I}(x,t) \end{array} \right ] +\left [ \begin{array}{@{}c@{}} (c_{1}/\tau_{E} )\hat{\xi}_{1}(x,t)\\ (c_{2}/\tau_{I} )\hat{\xi}_{2}(x,t) \end{array} \right ], \end{aligned} $$

with $\tilde {\mathbf{J}}(q)$ defined as in Eq. (15) with assumption that $\varLambda_{II}=0$, i.e., there is no self-inhibition in the system. We make Eq. (18) amenable to theoretical analysis by reformulating it into a two-variable Ornstein–Uhlenbeck (O–U) system of equations using stochastic techniques described by Chaturvedi et al. [31] and Gardiner [32],

19 $$ \begin{aligned} \frac{\partial}{\partial t} \left [ \begin{array}{@{}c@{}} \hat{E}(x,t) \\ \hat{I}(x,t) \end{array} \right ]= -\tilde {\mathbf{A}}(q)\left [ \begin{array}{@{}c@{}} \hat{E}(x,t) \\ \hat{I}(x,t) \end{array} \right ]+\sqrt{\mathbf{D}}\left [ \begin{array}{@{}c@{}} \hat{\xi}_{1}(x,t)\\ \hat{\xi}_{2}(x,t) \end{array} \right ], \end{aligned} $$

where $\tilde {\mathbf{A}}=-\tilde {\mathbf{J}}$ is the *q*-dependent *drift matrix* and $\bf D$ is a diagonal $2\times2$*diffusion matrix*,

20 $$ \begin{aligned} \bf D=\left [ \begin{array}{@{}c@{\quad }c@{}} (c_{1}/\tau_{E})^{2}&0 \\ 0&(c_{2}/\tau_{I})^{2} \end{array} \right ]. \end{aligned} $$

Ornstein–Uhlenbeck stationary statistics is well documented [31, 32], allowing us to immediately write down expressions for spatial power spectral density, autocorrelation, and variance of noise-induced fluctuations in cortical firing rates.

#### Spatial Power Spectral Density

The *covariance* matrix for our two-dimensional system is defined

21 $$\begin{array}{rl} \tilde{G}\left(q,q^{\prime }\right) & =\lim_{t\to \infty }\left[\begin{array}{cc} \langle E(q,t)E(q^{\prime },t)\rangle & \langle E(q,t)I(q^{\prime },t)\rangle \\ \langle I(q,t)E(q^{\prime },t)\rangle & \langle I(q,t)I(q^{\prime },t)\rangle \end{array}\right]\\ & =2\pi \delta \left(q+q^{\prime }\right)\tilde{G}(q), \end{array}$$

where $\langle\cdot \rangle$ signifies the expected value, and $\tilde {\mathbf{G}}(q)$ is the spatial power spectrum computed as [31, 32]

22 $$ \tilde {\mathbf{G}}(q)=\frac{\det(\tilde {\mathbf{A}})\mathbf{D}+[\tilde {\mathbf{A}} -\operatorname {tr}(\tilde {\mathbf{A}})\mathbf{I}]\mathbf{D}[\tilde {\mathbf{A}}-\operatorname {tr}(\tilde {\mathbf{A}})\mathbf{I}]^{\mathrm{T}}}{2\operatorname {tr}(\tilde {\mathbf{A}})\det(\tilde {\mathbf{A}})}, $$

in which $\bf I$ is the 2×2 identity matrix; $\det(\cdot)$ and $\operatorname {tr}(\cdot)$ are the determinant and trace operators, respectively. To determine $\tilde {\mathbf{G}}(q)$ at a nominated subthreshold steady state and wavenumber *q*, we evaluate the Jacobian $\tilde {\mathbf{J}}$ of Eq. (15) at that fixed point, then substitute $\tilde {\mathbf{A}} = -\tilde {\mathbf{J}}$ into Eq. (22). The spatial power spectral density of the excitatory firing rate *E* at wavenumber *q* is then given by $[\tilde {\mathbf{G}}(q)]_{11}$, the $(1,1)$ entry of the $2\times2$ spectrum matrix.

The spatial variance of the *E*–*E* fluctuations is estimated by evaluating the integral $\int_{-\infty}^{\infty}[\tilde {\mathbf{G}}(q)]_{11}\,dq$; meanwhile the spatial autocorrelation of these fluctuations is extracted from the inverse Fourier transform, $[\mathbf{G}(x)]_{11} = \mathcal{F}^{-1}[\tilde {\mathbf{G}}(q)]_{11}$.

#### Temporal Autocorrelation and Variance

Following [31] and [32], one can express the temporal correlation matrix $\tilde {\mathbf{T}}$ as the product of matrix exponential $\exp(-\tilde {\mathbf{A}}(q)\cdot\tau)$, evaluated at lag *τ*, with spatial spectral density $\tilde {\mathbf{G}}(q)$:

23 $$ \tilde {\mathbf{T}}(q, \tau)={\exp\bigl(-\tilde {\mathbf{A}}(q)\cdot\tau \bigr)} \tilde {\mathbf{G}}(q),\quad \tau>0, $$

with symmetry property $\tilde {\mathbf{T}}(q, -\tau)=[\tilde {\mathbf{T}}(q, \tau)]^{T}$. The $[\tilde {\mathbf{T}}(q,\tau)]_{11}$ element gives the theoretical expression for *q* and *τ* dependent autocorrelation function. We calculate the theoretical temporal autocorrelation at discrete $\tau =\tau_{i}$ values as

24 $$ \int_{0}^{q_{\max}} \tilde {\mathbf{T}}(q, \tau=\tau_{i})\,dq=\mathbf{C}(\tau_{i}), $$

to produce the temporal autocorrelation function $\mathbf{C}(\tau)$. In order to compare theoretical predictions with numerical results, we build a *q* vector based on spatial characteristics of 1-D network. The maximum spatial frequency is $q_{\max}=N_{x}/{L}=1/\Delta x$ where $N_{x}$ is the number of grid points, Δ*x* is the spatial resolution of the 1-D rod and *L* is its length, setting the spacing in the *q*-domain as $\Delta q = {1}/{L}$.

The temporal variance is the value of temporal autocorrelation at $\tau = 0\mbox{ ms}$.

### Numerical Solution of the Stochastic W–C Equations

The stochastic differential equations were expressed as integro-differential equations (2) with the addition of the small spatio-temporal white noises of Eqs. (16). The four spatial convolutions (two per equation) were computed directly using Matlab’s cconv (circular convolution) function to implement periodic boundaries. For example, the convolution of the *E*–*E* connectivity kernel $w_{EE}(x)$ with $E(x,t)$, the excitatory activity along the cortical rod at time *t*,

$$ w_{EE}(x) \otimes E(x,t) \equiv \int_{-L/2}^{L/2} w_{EE}\bigl(x'\bigr) \cdot E\bigl(x-x',t \bigr)\,dx', $$

can be coded in vectorized Matlab form as

$$ \mathtt{ifftshift}(\mathtt{cconv}(\vec{w}_{EE}, \vec{E}, N_{x})*\Delta x), $$

where $L = N_{x} \Delta x$ is the length of the cortical rod sampled with resolution Δ*x* at $N_{x}$ points from $-L/2$ to $L/2$, $\vec{w}_{EE}$ is the vector of *E*–*E* kernel weights (Eq. (4)), and $\vec{E}$ is the vector of *E*-activity at time *t* along the rod; both vectors contain $N_{x}$ elements. The cconv output requires an ifftshift adjustment (swapping of left and right halves) to ensure that the location of the rod center at $x = 0$ is conserved by the circular convolution.

A fixed time-step Euler algorithm was used to integrate the equations: we used $\Delta t = 0.1\mbox{ ms}$ for saddle-node analysis; 0.005 ms for other instabilities. Initial values of the *E* and *I* firing activity were set to the steady states values computed numerically from the intersection of the excitatory and inhibitory nullclines. Simulation durations were 10, 1.0, 5.0, and 0.5 s for saddle-node, Hopf, Turing and Turing–Hopf bifurcations, respectively. Spatial resolution was Δx=1.5 μm for all simulations. Field lengths of $L=3, 1, 6, 6\mbox{ mm}$ were used for simulation of saddle-node, Hopf, Turing, and Turing-Hopf bifurcations respectively. Appendix B contains demonstration code.

## Results

In this section we describe the variety of distinct bifurcations accessible to a deterministic Wilson–Cowan model, then run a series of numerical simulations of the stochastic W–C model placed close to bifurcation. We analyze the noise-induced subthreshold fluctuation statistics, and compare the numerical results with linear Ornstein–Uhlenbeck theoretical predictions, showing excellent agreement. To demonstrate relevance to real neuroscience, we report some new results from our electrophysiology laboratory showing precursor electrical activity in slices of rodent brain-tissue that have been chemically treated to generate infrequent spontaneous seizures.

### Bifurcations Accessible to the Deterministic 1-D Wilson–Cowan Model

Four instability bifurcations of the deterministic Wilson–Cowan cortex are demonstrated here: saddle-node, Hopf, Turing, and interacting Turing–Hopf; and in the section following, we will explore and quantify the noise-induced fluctuation responses of the cortex during close approach to each bifurcation point. Note that the *oscillatory Turing* instability (also known as a *wave instability*) is missing from this list of accessible bifurcations; this is because our implementation of the W–C model contains only two interacting components (*E* and *I*), while a minimum of three components are needed to support emergence of traveling or standing waves [33–35]. We return to this point briefly in the Discussion.

#### Saddle-Node

A *saddle-node bifurcation* occurs when the midbranch of the S-bend curve of steady states collides with the bottom branch and annihilates at $P_{\mathrm{SN}}\simeq1.7892426576\mbox{ mV}$: see Fig. 1(a). This bifurcation results in the disappearance of a pair of steady states, forcing the system to move to an alternate state resulting in a qualitative change in system dynamics.

#### Hopf

A *Hopf bifurcation* occurs when the real part of the complex eigenvalue pair changes sign at $P_{\mathrm{HB}}\simeq2.1971513755\mbox{ mV}$ as indicated in Fig. 1(b), (c). This bifurcation also changes the qualitative behavior of the cortex, leading to emergence of temporal oscillations.

#### Turing

The dispersion curves for $P=2.34\mbox{ mV}$ are plotted in Fig. 2(a). Boosting the inhibitory synaptic range constants $\sigma_{EI}$ and $\sigma_{IE}$ elevates the dispersion curves. When $\sigma_{EI,IE}=200\text{ μm}$, parts of the *α*-curve exhibit positive excursions predicting the formation of spatial *Turing* structures with spatial frequency $q/2\pi\simeq1.6\mbox{ waves/mm}$. The time–space graph of Fig. 2(b) shows the simulation results from the corresponding stochastic model. Initialized at the homogeneous deterministic steady state, the network spontaneously evolves into a spatially periodic structure. Although the pattern first emerges as a small sinusoidal oscillation in space whose frequency matches that of the dominant mode, the pattern rapidly becomes strongly non-sinusoidal as it grows, with nonlinear contributions from a broad range of wavenumbers ($1.1 \lesssim q/2\pi \lesssim3\mbox{ mm}^{-1}$). We note that linear eigenvalue analysis is only valid while the fluctuations remain small and sinusoidal, and it cannot predict the form of the fully evolved nonlinear spatial structure.

#### Turing–Hopf

The *Turing–Hopf* spatio-temporal instability results from the interaction between Turing and Hopf bifurcations, so requires that the conditions for both bifurcations be met simultaneously—i.e., the system should be close to an HB point, and the corresponding *α* dispersion curve should have a positive excursion at some nonzero *q*-value. We select $P=2\mbox{ mV}$ to put the system in an unstable Hopf mode (green circle in Fig. 3(a)), and we set $\sigma_{EI,IE}=112\text{ μm}$ to induce a Turing instability (positive *α* for $q\neq0$). The red curve of Fig. 3(b) predicts a global ($q=0$) mode of temporal frequency $f\simeq47\mbox{ HZ}$. We simulate the corresponding stochastic model and plot the spatio-temporal graphs of the 1-D cortex in Fig. 3(c) (bird’s-eye view) and (d) (3-D view) showing the Turing–Hopf interaction. The temporal Hopf oscillations dominate first, then the Turing pattern emerges, leading to development of mixed-mode oscillations.

In the following sections we drive the subthreshold system toward distinct bifurcation points, limiting ourselves to small noise-induced subthreshold fluctuations that approach, but do not cross, bifurcation threshold. We focus on the altering spectral characteristics of these fluctuations, looking for evidence of *critical slowing*.

### Close Approach to State Transition in the Stochastic W–C Model

**Fig. 4:**
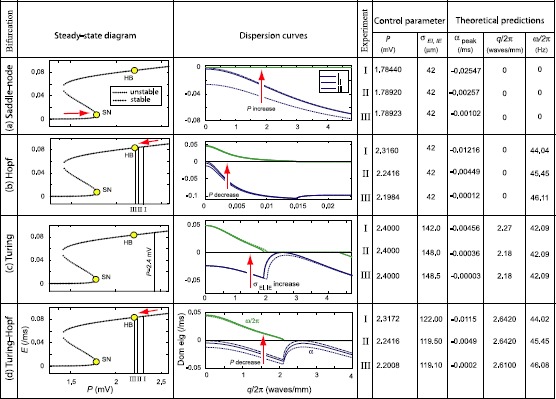
Approach to state transition in 1-D Wilson–Cowan cortex. The cortex is placed in subthreshold mode I, close to one of four bifurcation types, then driven toward instability in two steps (II, III) using one of two control parameters or a combination of both (*red arrows*). The resulting dispersion curves predict the spatial or temporal frequency of upcoming instabilities, determined by value of $q/2\pi$ at the peak of *α*-curve (*blue*) and the $\omega/2\pi$ value (*green*) at the $q=0$ axis, respectively

**Fig. 5:**
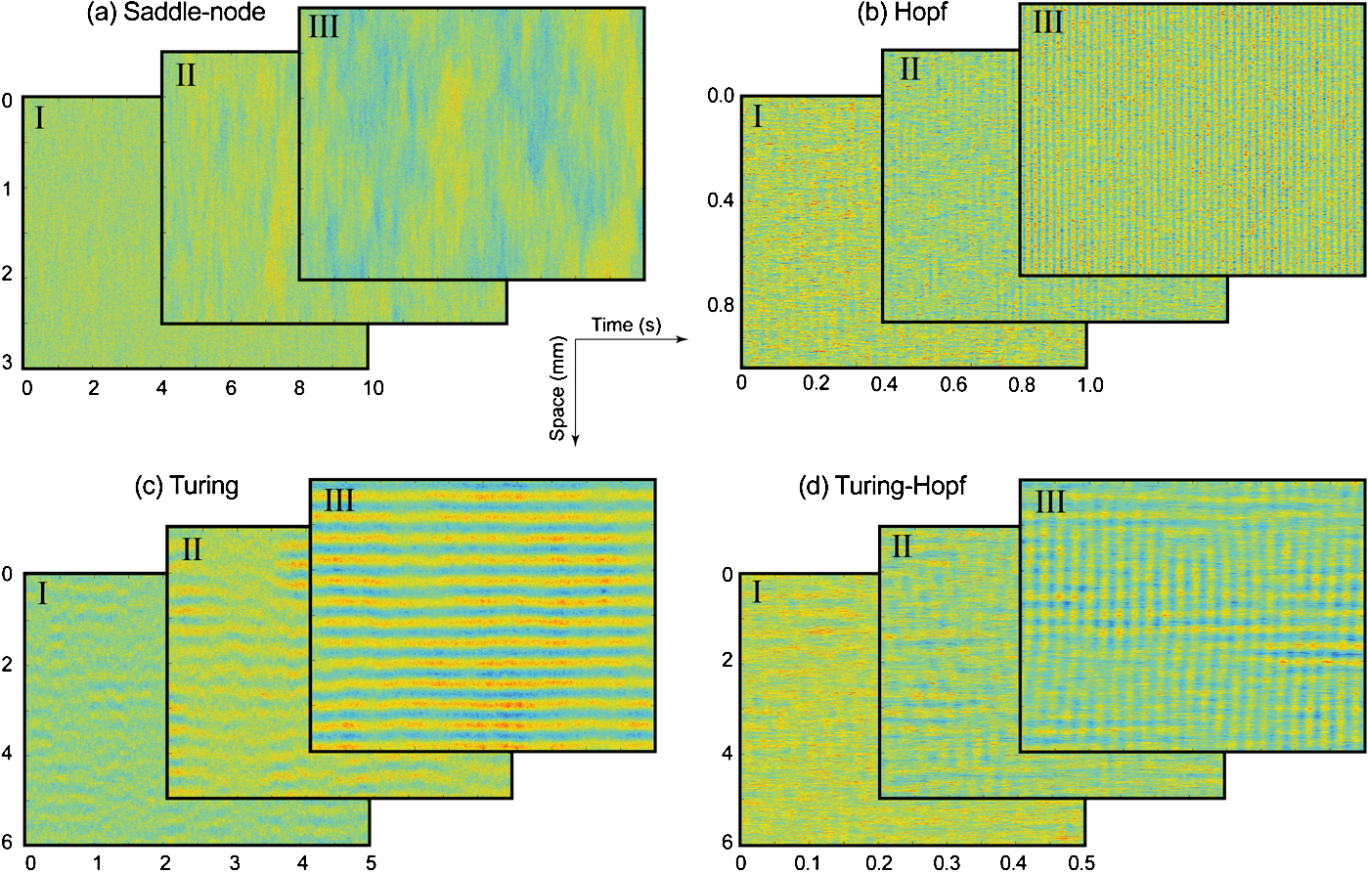
Growth of correlated fluctuations in time and/or space prior to state transition. The approach to the four distinct bifurcation classes is controlled via the parameters listed in Fig. 4. The results for each of the four subthreshold experiments are displayed in three layers *I–III*, with the topmost layer being closest to instability

**Fig. 6:**
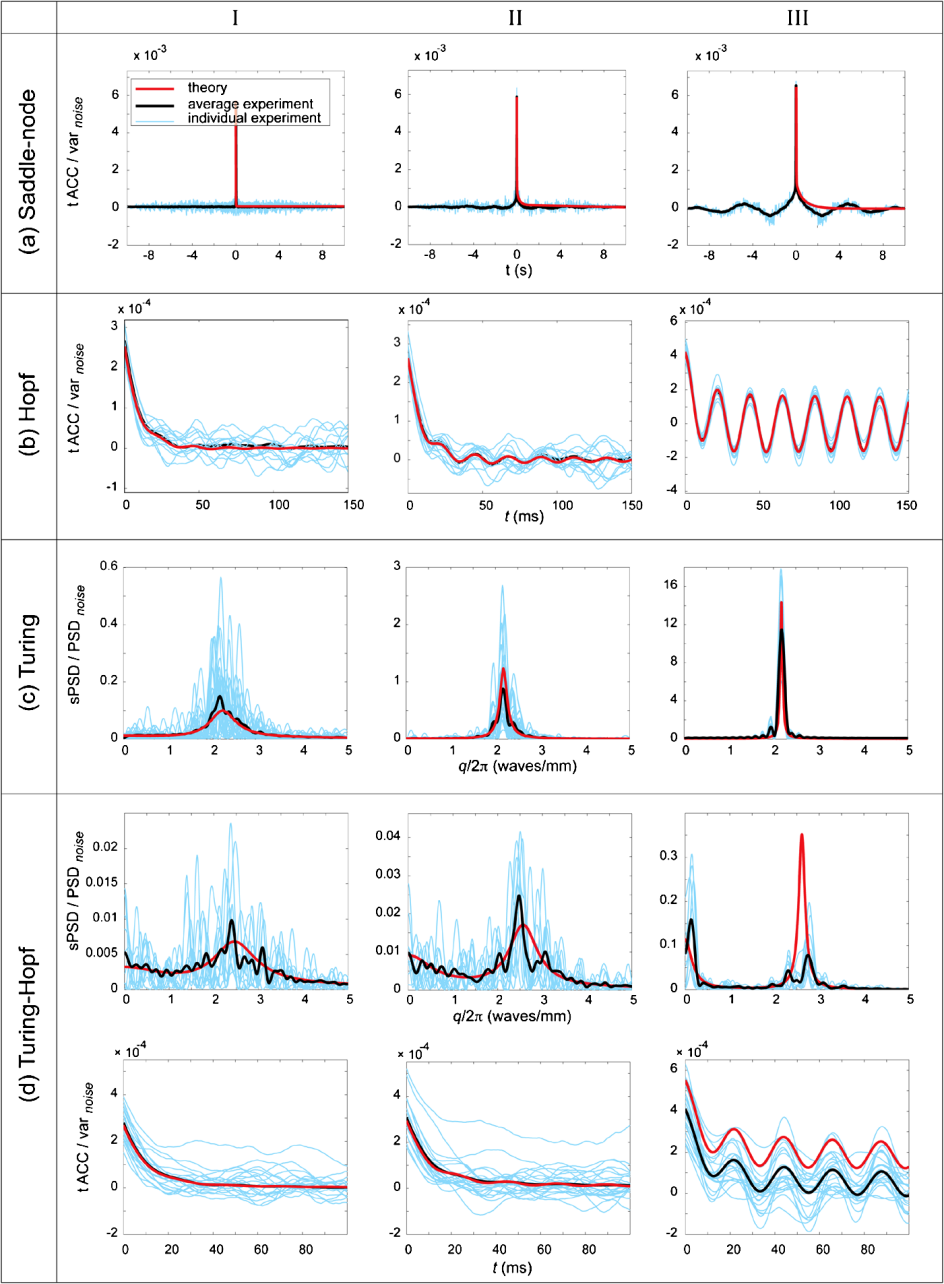
Temporal and spectral precursors of a phase transition. Noise-induced fluctuations of Wilson–Cowan 1-D cortex is studied via the temporal autocorrelation (tACC) and the spatial power spectral density (sPSD). Numerical simulation results (*black*) are compared with theoretical predictions (*red*) assuming Ornstein–Uhlenbeck stochastics following Gardiner [32]. Approach to **(a)** saddle-node bifurcation; **(b)** Hopf instability; **(c)** Turing instability; **(d)** mixed-mode oscillations

The results of theoretical and numerical examination of the stochastic 1-D Wilson–Cowan model prior to onset of its four bifurcation types are presented in this section. We sample the fluctuation characteristics of the model at three distinct steady-state coordinates, labeled I, II, III in Figs. 4–6, representing a closer and closer approach to instability threshold.

#### Homogeneous Steady States and Dispersion Curves

The steady state diagrams and dispersion curves of the 1-D cortex prior to bifurcation are displayed in Fig. 4. The subthreshold progress toward instability is indicated by the *α*-curves approaching zero from below. For the different bifurcation types, either one or two *α*-curve peaks are observed:

- A peak at the $q=0$ axis can be predictive of saddle-node or Hopf, with expected temporal frequency that can be read from the $\omega /2\pi$-curve.
- A peak at $q/2\pi\neq0$ suggests a Turing bifurcation at this nonzero spatial frequency.
- The presence of two peaks, one at $q/2\pi=0$ and the other at $q/2\pi\neq0$, predicts a mixed-mode Turing–Hopf spatio-temporal pattern.

The dispersion curves predict a “dc-resonant” frequency in space and time for saddle-node mode (Fig. 4(a)), while the approach to Hopf bifurcation is accompanied with temporal oscillations of frequencies $\omega/2\pi=44.04, 45.45, 46.11\mbox{ Hz}$ (Fig. 4(b)). Spatial frequencies of $q/2\pi=2.27, 2.18, 2.18\mbox{ waves/mm}$ are predicted for neural activity along the cortical rod when Turing instability is approached (Fig. 4(c)). Note that the strong dominance of Turing over Hopf instability (compare peak values of *α*-curve at $q=0$ axis and at $q/2\pi\simeq2.7\mbox{ waves/mm}$) suppresses the formation of temporal oscillations in Turing mode. In contrast, the dispersion curves in Fig. 4(d) feature dual *α*-curve peaks of comparable heights, suggesting the emergence of mixed-mode oscillations with frequencies of $\omega/2\pi=44.02, 45.145, 46.08\mbox{ Hz}$ and $q/2\pi \simeq2.64, 2.64, 2.61\mbox{ waves/mm}$ in time and space, respectively.

#### Numerical Study and Ornstein–Uhlenbeck Predictions

Using the values for control parameters of Fig. 4, we performed three sets of stochastic simulations for each of the four bifurcations. Results are summarized in Fig. 5(a–d), showing the spatio-temporal evolution of excitatory firing rate (*E*) of cortical neurons as a function of time and space. The emergent patterns are distinctive to each bifurcation class. Proximity to saddle-node is characterized by low-frequency (asymptotically zero frequency) fluctuations in both time and space (Fig. 5(a)), while temporal oscillations in the form of vertical stripes in Fig. 5(b) are indicative of an upcoming Hopf instability. The noise-induced fluctuations of the system arrange themselves in space as horizontal strips in Fig. 5(c) when the system is advanced toward the Turing threshold. Placing the system in the vicinity of simultaneous Hopf and Turing instabilities results in mixed-mode oscillations in time and space (Fig. 5(d)). In all cases, the patterns strengthen and become more distinct on close approach to the bifurcation point.

A quantitative analysis of simulation results is performed by computing the temporal autocorrelations (tACC) (for saddle-node, Hopf, and mixed-mode instabilities) and spatial power spectral density (sPSD) (for Turing and mixed-mode instabilities). In Fig. 6 we compare stochastic simulations with linear Ornstein–Uhlenbeck statistical projections for both tACC and sPSD, expressing the results as ratios relative to the variance and spectral density of the white noise stimulus. For each bifurcation type, the three graphs correspond to the three layers of Fig. 5.

For a saddle-node, the experimental tACC is the average of temporal autocorrelations of noise-induced *E* fluctuations obtained from 300 elements evenly distributed along the cortical rod (some of these traces are superimposed in Fig. 6(a)). The theoretical tACC (thick red curve) shows good agreement with experiment. We observe a pronounced widening of the autocorrelation curve, indicating a strong increase in correlation time, as the saddle-node annihilation point is approached. The unexpected appearance of side-lobe oscillations in the experimental tACC curves is an artifact caused by the finite length of the rod and the finite duration of the simulations: these “oscillations” damp out for longer rod lengths and simulation durations. A small growth in temporal variance (equal to tACC value at the origin) is also evident.

Theoretical and experimental temporal autocorrelations of *E*-fluctuations close to the Hopf bifurcation are displayed in Fig. 6(b). Growth in the amplitude of tACC at the origin and the side lobes is evident as the system is driven toward the Hopf instability. The experimental autocorrelation function is computed as the average of individual tACCs (thin blue traces) from 100 sample points evenly distributed along the cortical rod. The experiments confirm the emergence and amplification of temporal oscillations of frequency $f\simeq46\mbox{ Hz}$.

We analyze the spatial patterns of Turing and mixed-mode oscillations in terms of the spatial spectral density of the *E*-fluctuations. The sPSD (theoretical and experimental) for the Turing instability is plotted in Fig. 6(c). Here we have used the *E*-values of the entire 1-D rod at 10,000 instants evenly distributed between $t=0$ and $t=5\mbox{ s}$ to compute an average experimental sPSD. We see that approach to the Turing threshold is accompanied by a significant increase in the peak value of sPSD at spatial frequency $q/2\pi\simeq2.2\mbox{ waves/mm}$, as predicted in the dispersion curves of Fig. 4(c).

We plot the theoretical and experimental sPSD for mixed-mode oscillations in Fig. 6(d). As expected from Fig. 4(d), twin peaks emerge at $q=0$ and $q/2\pi\simeq2.6\mbox{ waves/mm}$, and these grow on approach to the threshold of the Turing–Hopf instability. The temporal dynamics prior to the mixed-mode instability is also quantified in this figure using tACC of the *E*-fluctuations. The results are similar to the Hopf case, showing an increased amplitude of tACC at the origin and the side lobes with temporal frequency $f\simeq 45\mbox{ Hz}$. Note the discrepancy between theoretical and experimental results when the state is very close to the threshold of the mixed-mode instability. We propose that this discrepancy arises from nonlinear interactions between the two types of bifurcation, making linear stability predictions less accurate.

**Fig. 7:**
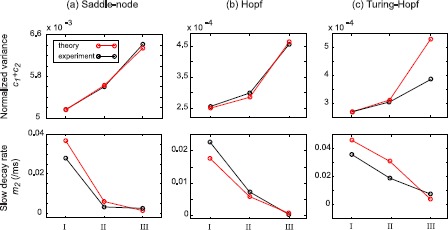
Growth of zero-lag temporal autocorrelation and slowing of decay-rate prior to phase transition. Biexponential expressions of the form $c_{1} \exp(-m_{1} \tau) + c_{2} \exp(-m_{2} \tau)$ were fitted to the decay envelopes of the temporal autocorrelations of Fig. 6. *Top panels* display predicted and measured normalized variance $c_{1} + c_{2}$ of the fitted curve; *bottom panels* are the $m_{2}$ slow exponential decay-rates (in (ms)^−1^)

To quantify the growth in correlation times, we fitted biexponential expressions to the envelope of temporal autocorrelation functions of Fig. 6,

$$ c_{1} \exp(-m_{1} \tau) + c_{2} \exp(-m_{2} \tau), \quad \text{with } m_{1} > m_{2} > 0, $$

representing the sum of fast ($m_{1}$) and slow ($m_{2}$) exponential decays. The sum $(c_{1}+c_{2})$ estimates the fluctuation variance (zero-lag autocorrelation), and $m_{2}$ gives the slow decay-rate (inverse correlation time). Plotted in the top and bottom panels of Fig. 7, respectively, these graphs show the expected indicators of critical slowing: growth of fluctuation variance and duration as bifurcation is approached.

**Fig. 8:**
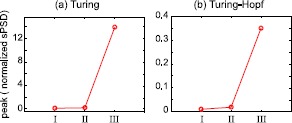
Growth of spatial spectral power prior to phase transition. Peak value of theoretical spectra of Fig. 6 are plotted here for **(a)** Turing, and **(b)** Turing–Hopf bifurcations

We also plotted the peaks of the theoretical spatial power spectral density functions of Fig. 6 for the Turing (see Fig. 8(a)) and the Turing–Hopf (Fig. 8(b)) cases. The increased power on approaching to bifurcation indicates a concentration of spectral content in an increasingly focused frequency range.

### *In Vitro* Evidence for Critical Slowing Near Bifurcation in a Brain Slice

The stochastic Wilson–Cowan model runs, supported by linear Ornstein–Uhlenbeck analysis, are unambiguous in their predictions: *irrespective of bifurcation type*, noise-induced fluctuations are expected to increase in intensity while becoming more prolonged in space and time (i.e., become critically slowed) during close approach to the bifurcation point. Thus, near a given critical point, we can expect specific universal characteristics to emerge in the statistical properties of the fluctuations. But these mathematical predictions are based on an idealized cortex—does the notion of criticality have any significance for real biological organisms in general and for neuroscience in particular?

To address this question, we have closely examined the spontaneous electrical activity in slices of mouse-brain tissue (from the hippocampus) perfused by an artificial cerebral spinal fluid containing zero concentration of magnesium ions (this causes NMDA channels to open) to which is added a low concentration of carbachol (to activate acetylcholine receptors) (see Ch. 7 of [36] for details). This preparation encourages spontaneous formation of transient avalanches of electrical activity in the slice, which have been likened to epileptic seizures, so they are referred to as seizure-like events (SLEs). If the emergence of an SLE represents a transition through a neural bifurcation point, then one would expect to see evidence of criticality in the local field potentials in the period leading up to the event.

**Fig. 9:**
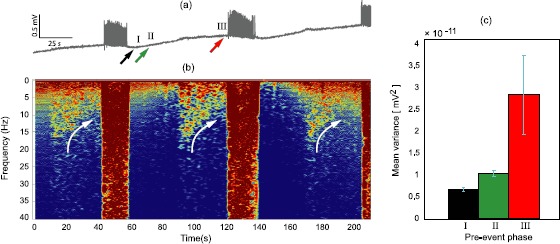
*In vitro* evidence of critical slowing near onset of a phase transition. **(a)** Local field potential recordings showing three spontaneous seizure-like events (SLEs) in a slice of mouse-brain tissue. **(b)** Spectrograms show characteristic “down-chirp” (drift to lower frequencies) in spectral activity as SLE onset is approached (*white arrows*). Note that the frequency scale increases vertically downwards. **(c)** The quiescent interval between consecutive events is sampled for 1 s at three representative times (labeled *I*, *II*, *III*) for variance analysis. Bars show the variance distribution across 40 inter-SLE periods. Note the pronounced growth in variance as the moment of seizure onset is approached

Figure 9(a) shows three consecutive SLEs that are well separated in time: each avalanche lasts ∼20 s, with about 60 s of electrical quiescence between events. Close examination of each quiescent interval reveals a subtle growth in background activity that commences about 30 s prior to avalanche, and that this activity has spectral energy that initially extends to ∼20 Hz, but drops to lower frequencies as onset approaches, forming a characteristic “down-chirp” in the spectrogram of Fig. 9(b). The 1-s fluctuation variances at three representative times (labeled I, II, III in panel (a)) show pronounced growth in background activity as the transition point is approached.

These observations provide pleasing qualitative support for the conjecture that seizure can be modeled as a neural bifurcation showing precursive characteristics of critical slowing and growing of fluctuation activity.

## Discussion

This paper adopts a lightly modified form of the one-dimensional *E*–*I* Wilson–Cowan network equations as an ideal testbed for investigating subthreshold dynamics prior to state transition. We used linear stability analysis to predict activity patterns emerging from the stationary homogeneous state of the W–C cortex. With appropriate selection of parameters, we obtain different patterns associated with the different bifurcations: bistability (saddle-node), bulk oscillations (Hopf), stationary patterns (Turing), and drifting patterns (Turing–Hopf). We quantified the impact of spatio-temporal noise on the homogeneous steady state by performing a linearization to derive an effective spatially extended Ornstein–Uhlenbeck process, allowing us to approximate the spatial power spectral density and correlation functions for the stochastic system. Near the saddle-node, the autocorrelation curve widens, indicating an increase in correlation time. Near the Hopf case, the autocorrelation grows at the origin and side lobes associated with the oscillation frequency. Near the Turing bifurcation, the power spectrum grows at the bifurcation pattern frequency. Finally, near the Turing–Hopf case, both the power spectrum and the autocorrelation grow at the onsetting pattern’s chosen frequency.

Missing from this list of bifurcations of the W–C network is the wave instability arising from an oscillating Turing. This is because, in the absence of delays, it is not possible to generate an oscillating Turing in the two-variable W–C model: such an instability can only occur in reaction–diffusion systems of three or more morphogens. This was first pointed out by Turing in his foundational 1952 paper [33], and more recent papers have used this fact to extract mathematical conditions for the occurrence of a wave instability in three-component reaction–diffusion systems [34, 35].

Hutt et al. [27, 28] have shown that under certain specific conditions, the presence of noise can delay the onset of a Turing bifurcation in a neural field. The delay occurs if the noise is “global” (uncorrelated in time while constant in space) since it induces a form of space-locking of field activity; but no such delay was reported in the case of fully uncorrelated noise (i.e., noise which is white in both space and time). Because all of our numerical simulations use white noise that is completely *uncorrelated* in space and time, our experiments of Fig. 5(c) near the Turing critical point of Fig. 4(c) do *not* exhibit any onset delay, and thus the stochastic simulations for close approach to the Turing bifurcation for the 1-D cortex are in excellent agreement with the Chaturvedi et al. [31] Ornstein–Uhlenbeck predictions for white-noise-induced spatial power spectral densities (compare black and red traces of Fig. 6(c)).

The fact that we are able to predict accurately the *nonlinear* growth in system responsiveness using *linear* stochastic theory is both surprising and satisfying. The success of linear stochastics arises because the noise stimulus and the resulting fluctuation amplitudes are small, so the eigenvalues obtained from linear stability analysis provide a good characterization of the deterministic response to the train of stochastic impulses from the noise. The critical slowing near threshold is governed by the weakening decay-rate of the dominant eigenvalue: at the critical point, the decay-rate is zero, so the perturbation response becomes infinitely prolonged.

The observed growth and widening of temporal autocorrelations and amplification of power at specific frequencies near instability onset are characteristic of systems approaching phase transition. Dramatic switching of brain activity to a new state is observed in both healthy and pathological cases, for example during wake–sleep and wake–anesthesia cycles, and at seizure onset. It has been proposed recently by Jirsa et al. that different bifurcation types may be responsible for these neural state transitions [37]. Indeed, the paradoxical boost in neural activity prior to anesthesia-induced loss of consciousness [38] may be a manifestation of critical slowing prior to loss of consciousness [39, 40].

The translation of our Wilson–Cowan model predictions—namely critical slowing near bifurcation onset—into real clinical applications will not be trivial. Under the very strictly controlled conditions of the *in vitro* brain slice, we were only able to see clear frequency changes in our local field potential recordings (Fig. 9) after going to the utmost lengths to suppress experimental noise (such as electrode drift, electromagnetic interference, vibration-induced artifacts, ground loops). This is because the subthreshold fluctuations are orders of magnitude smaller than the huge nonlinear oscillations that erupt beyond the bifurcation point. Nevertheless, our model does indicate an alternative approach to seizure prediction: current attempts at seizure prediction have concentrated on the detection of very high frequency oscillations as seizure precursors [41]; we suggest that it might be profitable to look instead at changes in very low temporal and spatial frequencies as indicators of imminent seizure.

## Appendix A

### A.1 Verification of Flux Equations for 1-D Wilson–Cowan Model Eqs. (10)

Here we show that the excitatory and inhibitory fluxes entering the population of type *k*,

A.1 $$ \begin{aligned} \phi_{Ek}(x,t)&=\int_{-\infty}^{\infty} E\bigl(x',t\bigr) n_{Ek}\bigl(x-x'\bigr) \,dx',\\ \phi_{Ik}(x,t)&=\int_{-\infty}^{\infty} I \bigl(x',t\bigr) n_{Ik}\bigl(x-x'\bigr) \,dx',\quad k\in\{E,I\}, \end{aligned} $$

are a solution of the partial differential equations (10). Define the Fourier transforms

A.2 $$ \begin{aligned} \tilde{\phi}_{Ek}(q,t)&=\frac{1}{2\pi}\int _{-\infty}^{+\infty}dx\, \phi _{Ek}(x,t)\mathrm{e}^{-iqx}, \\ \tilde{E}_{Ek}(q,t)&=\frac{1}{2\pi}\int_{-\infty}^{+\infty}dx\, E_{Ek}(x,t)\mathrm{e}^{-iqx}, \end{aligned} $$

and the corresponding inverse mappings:

A.3 $$ \begin{aligned} \phi_{Ek}(x,t)&=\int_{-\infty}^{+\infty}dq\, \tilde{\phi}_{Ek}(q,t)\mathrm{e}^{iqx}, \\ E_{Ek}(x,t)&=\int_{-\infty}^{+\infty}dq \,\tilde{E}_{Ek}(q,t)\mathrm{e}^{iqx}. \end{aligned} $$

Since the first equation in Eq. (A.1) is a convolution, its Fourier domain equivalent is

A.4 $$ \tilde{\phi}_{Ek}(q,t)=2\pi\tilde{E}(q,t)\cdot \tilde{n}(q). $$

Substitute (A.2), (A.3) in the first equation of (10):

A.5 $$ \begin{aligned}[b] &\frac{1}{2\pi}\int_{-\infty}^{+\infty} \bigl(\varLambda_{Ek}^{2}+q^{2}\bigr)\tilde{\varPhi}_{Ek}(q,t)\mathrm{e}^{-iqx}\,dq\\ &\quad =\frac{1}{2\pi}\int _{-\infty}^{+\infty }\varLambda_{Ek}^{2}\tilde{E}(q,t)\mathrm{e}^{-iqx}\,dq. \end{aligned} $$

Equating integrands:

A.6 $$ \bigl(\varLambda_{Ek}^{2}+q^{2} \bigr)\tilde{\varPhi}_{Ek}(q,t)=\varLambda_{Ek}^{2}\tilde{E}(q,t), $$

using (A.4):

A.7 $$ \tilde{n}(q)=\frac{1}{2\pi} \biggl( \frac{\varLambda_{Ek}^{2}}{\varLambda _{Ek}^{2}+q^{2}} \biggr). $$

#### Note

From Eq. (4):

A.8 $$ \begin{aligned}[b] \tilde{n}(q)&=\frac{1}{2\pi} \biggl( \frac{\varLambda_{jk}}{2}\int_{-\infty }^{+\infty} dx \,\mathrm{e}^{-\varLambda_{jk}\vert x\vert }\mathrm{e}^{-iqx} \biggr) \\ &=\frac{\varLambda_{jk}}{2(2\pi)} \biggl\{ \int_{-\infty}^{0} dx\, \mathrm{e}^{\varLambda_{jk}x-iqx}+\int_{0}^{\infty} dx\, \mathrm{e}^{-\varLambda _{jk}x-iqx} \biggr\} \\ &=\frac{\varLambda_{jk}}{2(2\pi)} \biggl\{ \biggl[ \frac{\mathrm{e} ^{\varLambda _{jk}x-iqx}}{\varLambda_{jk}-iq} \biggr]_{-\infty}^{0} + \biggl[ \frac {\mathrm{e} ^{-\varLambda_{jk}x-iqx}}{-(\varLambda_{jk}+iq)} \biggr]_{0}^{+\infty } \biggr\} \\ &=\frac{\varLambda_{jk}}{2(2\pi)} \biggl[ \frac{1}{\varLambda_{jk}-iq} + \frac {1}{\varLambda_{jk}+iq} \biggr] \\ &=\frac{\varLambda_{jk}}{2(2\pi)} \biggl[\frac{\varLambda_{jk}+iq+\varLambda _{jk}-iq}{\varLambda_{jk}^{2}+q^{2}} \biggr]=\frac{1}{2\pi} \biggl(\frac{\varLambda _{jk}^{2}}{\varLambda_{jk}^{2}+q^{2}} \biggr), \end{aligned} $$

which is the same result as (A.7). Thus $n(x) = \frac{\varLambda_{Ek}}{2}\mathrm{e} ^{-\varLambda_{Ek}\vert x\vert }$ and $\varPhi_{Ek}(x,t)$ satisfy Eqs. (10).

## Appendix B

### B.1 Matlab Code

The Euler implementation of 1-D Wilson–Cowan model is presented in this appendix. The code demonstrates pre-Turing and pre-Hopf simulations (using TurHopf flag) in response to a delta function or continuously applied white noise (using ImpStoch flag). One can reproduce Figs. 5(b-III), (c-III) by setting ImpStoch=0.

Initial values for *E* and *I* firing rates were set to the steady state values extracted from intersection of model nullclines.

Lines 48–53 demonstrate how white noise is added to the initial steady-state values. To expedite Turing emergence, the network is energized with boosted noise at the first time step; the noise amplitude is attenuated (line 48) thereafter.

The fixed-step Euler method uses model derivatives (computed by the nested function EI_derivs on line 92) to update *E* and *I* activities. Spatio-temporally uncorrelated white noise is generated using Matlab’s randn function at every time step (lines 98, 99), but is inactivated (sf=0 for the impulse runs).

Very long simulations are needed (Last=1000, 5000 for pre-Hopf and pre-Turing cases, respectively) with very fine time steps (dt=0.005 ms) in order to obtain fully evolved patterns similar to Figs. 5(b-III) and (c-III).

## References

[1] Scheffer M, Carpenter SR, Lenton TM, Bascompte J, Brock W, Dakos V, van de Koppel J, van de Leemput IA, Levin SA, van Nes EH (2012). Anticipating critical transitions. Science.

[2] Carpenter S, Brock W (2006). Rising variance: a leading indicator of ecological transition. Ecol Lett.

[3] Dakos V, Scheffer M, van Nes EH, Brovkin V, Petoukhov V, Held H (2008). Slowing down as an early warning signal for abrupt climate change. Proc Natl Acad Sci USA.

[4] Ives AR (1995). Measuring resilience in stochastic systems. Ecol Monogr.

[5] Veraart AJ, Faassen EJ, Dakos V, van Nes EH, Lürling M, Scheffer M (2012). Recovery rates reflect distance to a tipping point in a living system. Nature.

[6] Carpenter SR, Cole JJ, Pace ML, Batt R, Brock W, Cline T, Coloso J, Hodgson JR, Kitchell JF, Seekell DA (2011). Early warnings of regime shifts: a whole-ecosystem experiment. Science.

[7] Zhang X, Zahn M (2014). Electro-optic precursors of critical transitions in dielectric liquids. Appl Phys Lett.

[8] van de Leemput IA, Wichers M, Cramer AO, Borsboom D, Tuerlinckx F, Kuppens P, van Nes EH, Viechtbauer W, Giltay EJ, Aggen SH (2014). Critical slowing down as early warning for the onset and termination of depression. Proc Natl Acad Sci USA.

[9] Matsumoto G, Kunisawa T (1978). Critical slowing-down near the transition region from the resting to time-ordered states in squid giant axons. J Phys Soc Jpn.

[10] Steyn-Ross DA, Steyn-Ross ML, Wilson MT, Sleigh JW (2006). White-noise susceptibility and critical slowing in neurons near spiking threshold. Phys Rev E.

[11] Wilson MT, Barry M, Reynolds JN, Hutchison E, Steyn-Ross DA (2008). Characteristics of temporal fluctuations in the hyperpolarized state of the cortical slow oscillation. Phys Rev E.

[12] Meisel C, Kuehn C (2012). Scaling effects and spatio-temporal multilevel dynamics in epileptic seizures. PLoS ONE.

[13] Steyn-Ross DA, Steyn-Ross ML, Wilson MT, Sleigh JW, Steyn-Ross DA, Steyn-Ross M (2010). Phase transitions in single neurons and neural populations: critical slowing, anesthesia, and sleep cycles. Modeling phase transitions in the brain.

[14] Destexhe A, Contreras D, Steriade M (1999). Spatiotemporal analysis of local field potentials and unit discharges in cat cerebral cortex during natural wake and sleep states. J Neurosci.

[15] Steyn-Ross ML, Steyn-Ross DA, Sleigh JW, Whiting D (2003). Theoretical predictions for spatial covariance of the electroencephalographic signal during the anesthetic-induced phase transition: increased correlation length and emergence of spatial self-organization. Phys Rev E.

[16] Wilson HR, Cowan JD (1972). Excitatory and inhibitory interactions in localized populations of model neurons. Biophys J.

[17] Wilson HR, Cowan JD (1973). A mathematical theory of the functional dynamics of cortical and thalamic nervous tissue. Kybernetik.

[18] Steyn-Ross DA, Steyn-Ross ML, Sleigh JW (2014). Equilibrium and nonequilibrium phase transitions in a continuum model of an anesthetized cortex. Neural fields.

[19] Ermentrout GB, Cowan JD (1979). A mathematical theory of visual hallucination patterns. Biol Cybern.

[20] Bressloff PC (2012). Spatiotemporal dynamics of continuum neural fields. J Phys A, Math Theor.

[21] Laing CR, Troy WC (2003). Two-bump solutions of Amari-type models of neuronal pattern formation. Phys D, Nonlinear Phenom.

[22] Coombes S, Laing C (2009). Delays in activity-based neural networks. Philos Trans R Soc, Math Phys Eng Sci.

[23] Hutt A (2013). The anesthetic propofol shifts the frequency of maximum spectral power in eeg during general anesthesia: analytical insights from a linear model. Front Comput Neurosci.

[24] Hindriks R, van Putten MJAM (2012). Meanfield modeling of propofol-induced changes in spontaneous EEG rhythms. NeuroImage.

[25] Robinson PA, Rennie CJ, Wright JJ, Bahramali H, Gordon E, Rowe DL (2001). Prediction of electroencephalographic spectra from neurophysiology. Phys Rev E.

[26] Hutt A, Frank T (2005). Critical fluctuations and 1/f *α*-activity of neural fields involving transmission delays. Acta Phys Pol A.

[27] Hutt A, Longtin A, Schimansky-Geier L (2008). Additive noise-induced turing transitions in spatial systems with application to neural fields and the Swift–Hohenberg equation. Phys D, Nonlinear Phenom.

[28] Hutt A, Steyn-Ross DA, Steyn-Ross M (2010). Spatiotemporal instabilities in neural fields and the effects of additive noise. Modeling phase transitions in the brain.

[29] Wilson HR (1999). Spikes, decisions, and actions: the dynamical foundations of neuroscience.

[30] Wang Y, Goodfellow M, Taylor PN, Baier G (2012). Phase space approach for modeling of epileptic dynamics. Phys Rev E.

[31] Chaturvedi S, Gardiner C, Matheson I, Walls D (1977). Stochastic analysis of a chemical reaction with spatial and temporal structures. J Stat Phys.

[32] Gardiner C (2004). Handbook of stochastic methods: for physics, chemistry and the natural sciences.

[33] Turing AM (1952). The chemical basis of morphogenesis. Philos Trans R Soc Lond.

[34] Anma A, Sakamoto K, Yoneda T (2012). Unstable subsystems cause Turing instability. Kodai Math J.

[35] Hata S, Nakao H, Mikhailov AS (2014). Dispersal-induced destabilization of metapopulations and oscillatory Turing patterns in ecological networks. Sci Rep.

[36] Negahbani E. Dynamics and precursor signs for phase transitions in neural systems. PhD thesis. Hamilton, New Zealand: University of Waikato; 2015.

[37] Jirsa VK, Stacey WC, Quilichini PP, Ivanov AI, Bernard C (2014). On the nature of seizure dynamics. Brain.

[38] Kuizenga K, Wierda J, Kalkman C (2001). Biphasic EEG changes in relation to loss of consciousness during induction with thiopental, propofol, etomidate, midazolam or sevoflurane. Br J Anaesth.

[39] Steyn-Ross ML, Steyn-Ross DA, Sleigh JW, Liley DTJ (1999). Theoretical electroencephalogram stationary spectrum for a white-noise-driven cortex: evidence for a general anesthetic-induced phase transition. Phys Rev E.

[40] Steyn-Ross ML, Steyn-Ross DA, Sleigh JW (2004). Modelling general anaesthesia as a first-order phase transition in the cortex. Prog Biophys Mol Biol.

[41] Worrell G, Gotman J (2011). High-frequency oscillations and other electrophysiological biomarkers of epilepsy: clinical studies. Biomark Med.

